# A Rare Presentation of an Isolated Radius Shaft Fracture in a Two-Month-Old Child: Dilemmas and Management

**DOI:** 10.7759/cureus.11947

**Published:** 2020-12-07

**Authors:** Thirumagal SK, Terrence Jose Jerome

**Affiliations:** 1 Trauma, Orthopaedics and Emergency, Hospital & Trauma Research Centre, Puthur High Road, Trichy, IND; 2 Orthopaedics, Hand and Reconstructive Microsurgery, Olympia Hospital and Research Centre, Trichy, IND

**Keywords:** dilemas, isolated, radius shaft, rare, two-month old child

## Abstract

In this report, we discuss the case of a two-month-old boy with an isolated radius shaft fracture in the right forearm. The history and nature of the injury may be inconclusive in such injuries. A radiograph confirmed that the child had a fracture. We treated the boy conservatively, and the fracture united well in four weeks. Surgeons should be aware of this rare presentation in infants of this age. Inquiry into possible child abuse as well as clinical and metabolic workup is essential in these cases.

## Introduction

Apart from obstetric fractures, it is rare for an orthopaedic surgeon to encounter fractures in children under six months of age [1]. A few reports have described 9% clavicular fractures in vaginal deliveries [1]. Proximal and distal humerus physeal injuries and femur and tibial fractures have been noted in children aged 18 months [2]. However, upper limb fractures in children in the age group of under six months are rare, and forearm fractures have not been reported in the literature. Diaphyseal fractures are easy to diagnose, but the challenge lies in recognizing physeal injuries in this age group. Identifying the aetiology is extremely difficult, and the treatment aspects will have medical, social and legal implications. Also, a multidisciplinary approach could benefit such children in facilitating their recovery and rehabilitation.

## Case presentation

**Figure 1:**
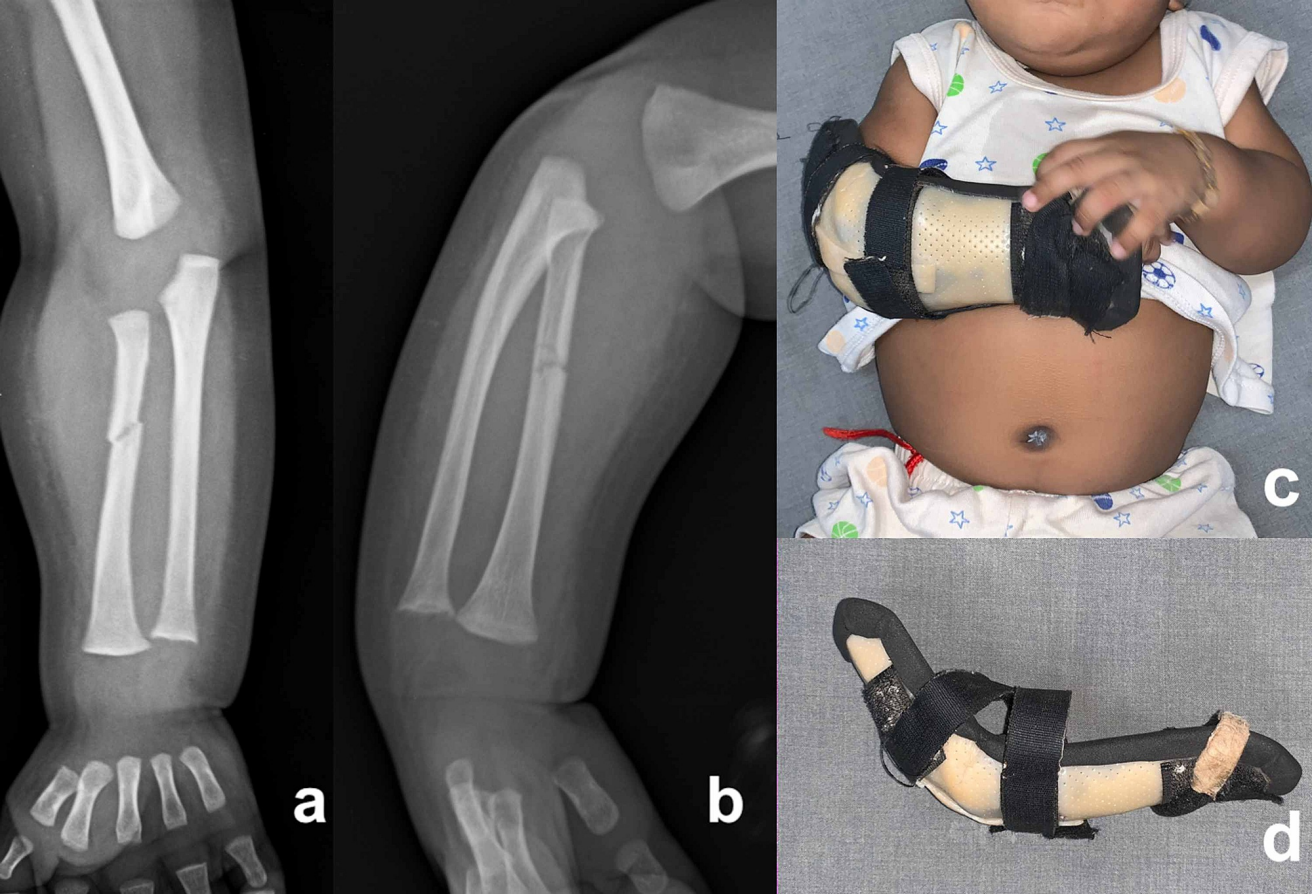
Radiographs at the time of diagnosis Radiographs of a two-month-old boy show isolated radius shaft fracture in posteroanterior (a) and oblique views (b). Clinical pictures of the boy (c) with an above-elbow thermoplastic splint (d) two weeks after the injury

We present a two-month-old healthy boy who was brought to the emergency room with a constant cry along with swelling and loss of active movement in the right upper limb. The patient's medical history was inconclusive, and we noted localized swelling in the proximal third of the forearm during the clinical examination. The proximal humerus, elbow and wrist passive movements were free and painless. There was no redness, bruises, burns or sinus over the swelling. We examined the left upper limb, both lower limbs and the rest of the body (Figure 1a, Figure 1b) and confirmed that there were no associated injuries.

**Figure 2:**
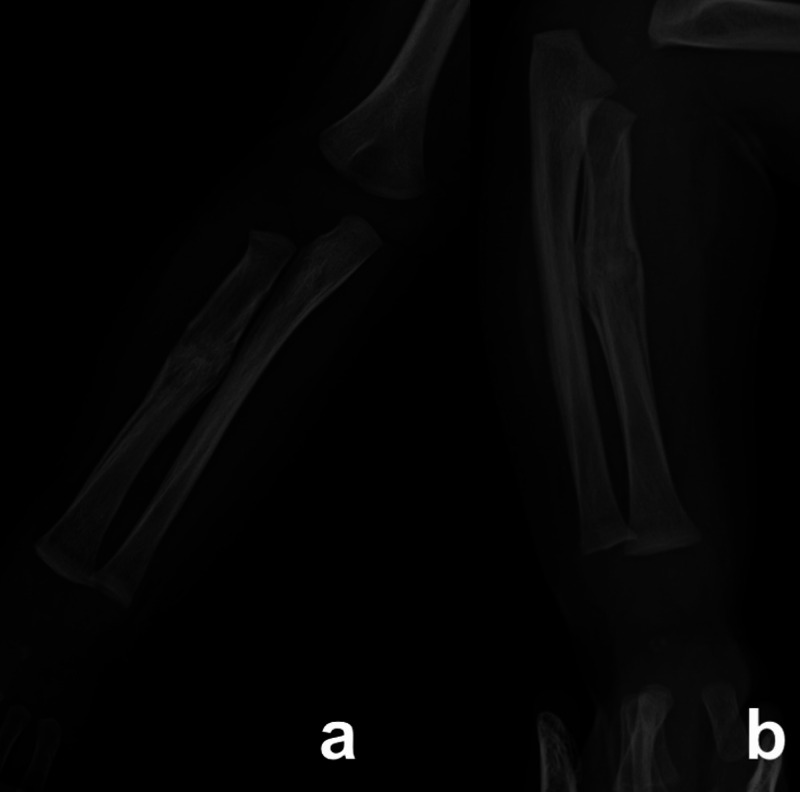
Radiographs at four weeks of treatment Radiographs show a well-united radius fracture in the posteroanterior (a) and oblique views (b)

The radiographs showed isolated radius midshaft fracture in the right forearm. We treated the child with an above-elbow slab and admitted him for overnight observation. The paediatrician performed a general and neurological evaluation of the child. The blood parameters were normal. We discharged the boy on the next day and advised the parents to keep vigil over the hand movements, discolourations and swelling. The boy tolerated the slab for a week with no associated complications. We removed the slab and applied an above-elbow thermoplastic splint for two weeks (Figure 1c, Figure 1d). The fracture united within four weeks (Figure 2). We removed the splint, which allowed activities of the right upper limb. 

## Discussion

The common causes of fractures in children under 18 months of age are accidental falls, child abuse, and abnormal bone fragility [1-3]. Our case presented to the emergency room with isolated forearm swelling and normal movements in the rest of the body. The diaphyseal location, painless elbow and wrist passive movement ruled out septic arthritis. Neglect or abuse of children is the second most common cause of injury in situations associated with soft tissue lesions, bruises, burns, face swelling, multiple limb swellings or damages to the brain and other inner organs [4]. The mean age in such cases ranges from six to 16 months [4]. Metaphyseal fractures, bilateral rib fractures, complex skull fractures, vertebral fractures and subluxations as well as finger fractures are common in the abused children [5]. Our patient was a hale and healthy boy with a unilateral forearm swelling, and the parents accompanying the child did not show any features associated with those who engaged in child abuse; there were no signs of battered baby syndrome either. Bone fragility syndromes include osteogenesis imperfecta, metaphyseal dysplasia, rickets, vitamin D deficiency, and temporary brittle bone disease. Unexplained fractures rarely occur in infantile/juvenile osteoporosis, osteopetrosis, leukaemia, hypophosphatasia, vitamin A and C deficiency and biliary atresia [6]. Normal blood parameters, absence of bilateral involvement, lack of bowing of limbs and abnormal appearance of metaphases (cupping, fraying and flaring), and the rare occurrence of bone fragility fractures before walking (18 months) distinguished our patient's isolated fracture in the forearm.

The management of fractures in neonates, infants and toddlers is usually conservative, which includes immobilization by bandages, cast, splinting, harness or traction. Fractures heal consistently and very rapidly. The outcome is usually good with bone union seen within three to four weeks for metaphyseal fractures and four to six weeks for diaphyseal fractures [1]. Despite the bone union in our case, we are still following up on the case closely. Eliciting corroborative history, multidisciplinary evaluation, and radiological surveys are indispensable for treating these fractures efficaciously.

## Conclusions

Surgeons should be aware of isolated fractures that can occur in children under six months of age. The fractures unite well in these children with adequate immobilization. Also, we should evaluate for accidental falls, child abuse and bone fragility syndromes with detailed clinical and metabolic workup for adequate management. The most critical aspect of avoiding or managing fractures in children of younger age lies in great care and prevention.
